# Nonenzymatic glycosylation of human serum albumin and its effect on antibodies profile in patients with diabetes mellitus

**DOI:** 10.1371/journal.pone.0176970

**Published:** 2017-05-17

**Authors:** Alok Raghav, Jamal Ahmad, Khursheed Alam

**Affiliations:** 1 Rajiv Gandhi Centre for Diabetes and Endocrinology,J.N. Medical College,Aligarh Muslim University, Aligarh, Uttar Pradesh, India; 2 Department of Biochemistry,Faculty of Medicine,J.N. Medical College,Aligarh Muslim University, Aligarh, Uttar Pradesh, India; Islamic Azad University Mashhad Branch, ISLAMIC REPUBLIC OF IRAN

## Abstract

**Background:**

Albumin glycation and subsequent formation of advanced glycation end products (AGEs) correlate with diabetes and associated complications.

**Methods:**

Human Serum Albumin (HSA) was modified with D-glucose for a 40 day period under sterile conditions at 37°C. Modified samples along with native HSA (unmodified) were analyzed for structural modifications by UV and fluorescence, FTIR, Liquid chromatography mass spectrometry (LCMS) and X–ray crystallography. New-Zealand white female rabbits immunized with AGEs, represent auto-antibodies formation as assessed by competitive and direct binding enzyme-linked immunosorbent assay (ELISA). Neo-epitopesagainst *In-vitro* formed AGEs were characterized in patients with diabetes mellitus type 2 (n = 50), type 1 (n = 50), gestational diabetes (n = 50) and type 2 with chronic kidney disease (CKD) with eGFR level 60–89 mL/min (n = 50) from serum direct binding ELISA.

**Results:**

Glycated-HSA showed amarked increase in hyperchromicity of 65.82%,71.98%, 73.62% and 76.63% at λ_280 nm_ along with anincreasein fluorescence intensity of 65.82%, 71.98%, 73.62% and 76.63% in glycated-HSA compared to native. FTIR results showed theshifting of Amide I peak from 1656 cm^_1^ to 1659 cm^_1^ and Amide II peak from 1554 cm^_1^ to 1564 cm^_1^ in glycated-HSA, with anew peak appearance of carbonyl group at 1737 cm-1. LCMS chromatogram of glycated-HSA showed thepresence of carboxymethyl lysine (CML) at 279.1 m/z. Immunological analysis showed high antibody titre>1:12,800 in theserum of rabbits immunized with glycated-HSA (modified with 400 mg/dL glucose) and inhibition of 84.65% at anantigen concentration of 20μg/mL. Maximum serum auto-antibody titre was found in T2DM (0.517±0.086), T1DM (0.108±0.092), GDM (0.611±0.041) and T2DM+CKD (0.096±0.25) patients immunized with glycated-HSA (modified with 400 mg/dL glucose).

**Conclusions:**

Non-enzymatic glycosylation of HSA manifests immunological complications in diabetes mellitus due to change in its structure that enhances neo-epitopes generation.

## Introduction

Glucose is a prime dietary component and energy exchange of the cell. Glucose, being a bio-molecule of great importance, promotes adverse metabolic alterations in biological systems that include hypoglycemia, glucose intolerance, glucotoxicity, and hypertension [1–5]. Diabetes mellitus is a hub of metabolic disorders represented by an imbalance in glucose homeostasis leading to impairment in carbohydrate, lipids, and protein metabolism [6]. It is well-established fact that prolonged exposure to proteins with sugars under hyperglycemia generates advanced glycation end products (AGEs) [7]. The non-enzymatic addition of sugar with protein initiates the cascade of pathological mechanism of diabetes and its associated micro- and macro-vascular complications [8]. Previous literature of immunohistochemical investigations demonstrates AGEs-altered proteins in various human tissues under pathological conditions, including the patients with diabetic nephropathy [9], cardiovascular disease [10] and diabetic retinopathy [11]. Few scientists also describe the role of *In-vivo* AGEs as immunological epitopes starting the generation of auto-antibodies that plays a role in the progression of immunological complications in diabetes mellitus and its associated complications [12]. AGEs formed *in-vivo* or *in-vitro* binds to their receptor known as a receptor for advanced glycation end product (RAGE) that belongs to transmembrane 35kDa protein IgG super-family that acts as a key player in immunological response [13]. RAGE is distributed in many cell types such as monocytes, endothelial cells, smooth muscle cells, hepatocytes, and neurons and their expression may significantly impair in diseased state [14–16]. Recent literature has shown that diabetic animal model expresses more RAGE compared to control animals [17].Glycation of protein impairs the secondary and tertiary structure leads to the development of neo-epitopes that have potential to bind with antibodies showing animmunological response.

With the onset of diabetes mellitus, persistent and prolonged chronic hyperglycemia enhances free radicals formation through auto-oxidation of aldehyde group of glucose via non-enzymatic glycation of proteins leading to increasing flux of glucose through polyol pathway [18]. There is aninterconnection between protein glycation and free radicals [19]. Reactive oxygen species (ROS) causes extensive deterioration in human serum albumin (HSA) structure along with other biomacromolecules which formneo-epitopes contributing to its immunogenic potential showed by several animal experiments and clinical studies in patients with diabetes mellitus and its associated complications [20–21]. Covalent attachment of glucose or small residues in autologous proteins and other bio-molecules can generate conjugates that are efficient in inducing an immune response in the host cells. Fabrication of specific glucose-derived adducts on biomacromolecules could function in a fashion to form auto-antibodies in diabetic patients. Qualitative studies have shown that chemically produced hexitolamino derivative of collagen Amadori adduct produces such Immunogenic response [22].Recent published literature demonstrated that non enzymation glycation of serum albumin leads to deterioration of albumin properties both structural and functional. Recently it is proved that human serum albumin during the course of non enzymatic glycation leads to development of advanced glycation end products that impairs its biochemical, electrochemical, spectroscopic, optical and fluidity properties [23]. Another study done with glycated HSA showed that presentation of neo-epitopes formed during the course of non enzymatic glycation due to refolding and structural impairments [24 –25].

The aim of this work was to investigate the immunological properties of AGEs formed at thephysiological glycemic and euglycemic range. Induced antibodies against *in-vitro* formed AGEs were implicated as a probe to detect antibody titer and impairment in homeostasis of thebiological system arising in diabetes and its associated complications.

## Material and methods

### Test materials and reagents

Human serum albumin (HSA), anti-rabbit IgG alkaline phosphatase conjugates, anti-human IgG alkaline phosphatase conjugates, pnitrophenyl phosphate, ethidium bromide, Tween-20, Protein A-agarose (2.5 mL pre-packed column), Freund’s complete and incomplete adjuvants, was purchased from Sigma chemical company (St. Louis, USA), D-glucose, sodium chloride (NaCl) Sodium mono phosphate, diphosphate was purchased from SRL chemicals, India. All others chemicals and reagents used were of highest analytical grade available.

### Ethics statement

Healthy female New Zealand white rabbits (1.5 ± 5 Kgs) were obtained from central animal facility and study was prospectively approved by Institutional animal ethics committee, Aligarh Muslim University, India (Certificate approval No. 401/RO/C/2001/CPCSEA) J.N Medical College, Aligarh Muslim University, India as per guideline laid down by CPCSEA (Ministry of Environment and Forests, Govt. of India). The study protocol does not include any anesthesia, euthanasia, or any kind of animal sacrifice. The protocol only involved exsanguination of blood from marginal ear vein at stipulated time intervals.

All subjects were informed about the study procedure and they provided written informed consent. The study was approved by the local Institutional Ethics Committee, Faculty of Medicine, J.N Medical College, Aligarh Muslim University, India (Certificate approval No. 1894/FM) (Govt. of India).

### Study design and patients recruitment

This immunological study includes a total of 400 subjects including T2DM (n = 50) (49±8.65 Years, age range 41–57 Years), T1DM (n = 50) (23.63±5.62 Years; age range 14–29 Years), GDM(n = 50) (31.23±9.56 Years; age range:22–40 Years), T2DM+CKD (n = 50) (58.22±6.45 Years; age range: 54–68 Years) patients along with healthy volunteers (n = 50)in each group who visited at Rajiv Gandhi Centre for Diabetes and Endocrinology, J.N Medical College, Aligarh Muslim University, Aligarh. The partcipants are recruited in the month of July to August, 2016. Venous fasting blood was collected from all subjects in vials for thecollection of blood serum and plasma.

### Inclusion criteria

[1] Type 2 Diabetes Mellitus (T2DM)

[2] Type 1 Diabetes Mellitus (T1DM)

[3] Gestational Diabetes (GDM)

[4] T2DM with chronic kidney disease (CKD) with eGFR values between 60–89 mL/min

### Exclusion criteria

[1] Subjects with any immunological complications.

### Preparation of advanced glycation end products (AGEs)

AGEs were prepared as previously mentioned protocol [26]. HSA (20 μM) was incubated under sterile conditions with varying concentrations of D-glucose (100, 200, 300 and 400 mg/dL) in phosphate buffer saline (20 mM, pH = 7.5) in the presence of 0.01% sodium azide solution at 37°C under sterile conditions in capped sterile tubes for 40 days. At the termination of incubation, the samples were extensively dialyzed against sterile PBS buffer (10mM, pH = 7.4) with consecutively two changes of PBS buffer overnight at 4°C to remove excess glucose. The samples were stored at -20°C for further analysis.

### Spectroscopic analysis

The ultraviolet absorption profile of native and modified HSA was scanned in the wavelength range of 190–400 nm on Shimadzu UV-1700 spectrophotometer with quartz cuvette having 1 cm path length. AGEs specific fluorescence of native and modified HSA sample was done on Shimadzu (RF-5301-PC) spectrofluorophotometer. The samples were excited at a wavelength of 370 nm, and the emission intensities were recorded at 400–600 nm range. To demonstrate the change in secondary structure upon glycation, Fourier transform-infra red spectroscopy analysis of native and HSA modified with 400 mg/dL concentration glucose was recorded. Briefly, 10 μl of protein sample was placed on ATR accessory on FT-IR spectrophotometer (8201 PC) with a resolution of 4 cm-1. FT-IR measurements of native and glycated samples were carried on Shimadzu FT-IR spectrophotometer (8201-PC) in the spectral range of 400–4000 cm^-1^.

### Liquid chromatography mass spectroscopic analysis

Native and glycated-HSA were subjected to LCMS analysis for identification of AGEs related adduct upon modifications along with carboxymethyl-lysine (CML), as the standard for AGEs. Samples were hydrolyzed with previously described protocol [27]. The digested hydrolyzate followed by filtration loaded into reverse phase separation assembly coupled with the capillary HPLC system having a C 18 analytical column. Briefly 0.4% acetic acid (solvent A), 0.2% acetonitrile (solvent B) comprising 2% formic acid were used as chromatographic conditions. The mass spectroscopic analysis was then performed on Micromass Castro Ultima Triple Quadrupole Mass spectrometer operated in positive ion mode with a full scan range from 0–400 m/z range.

### Immunization schedule

**Fig 1:**
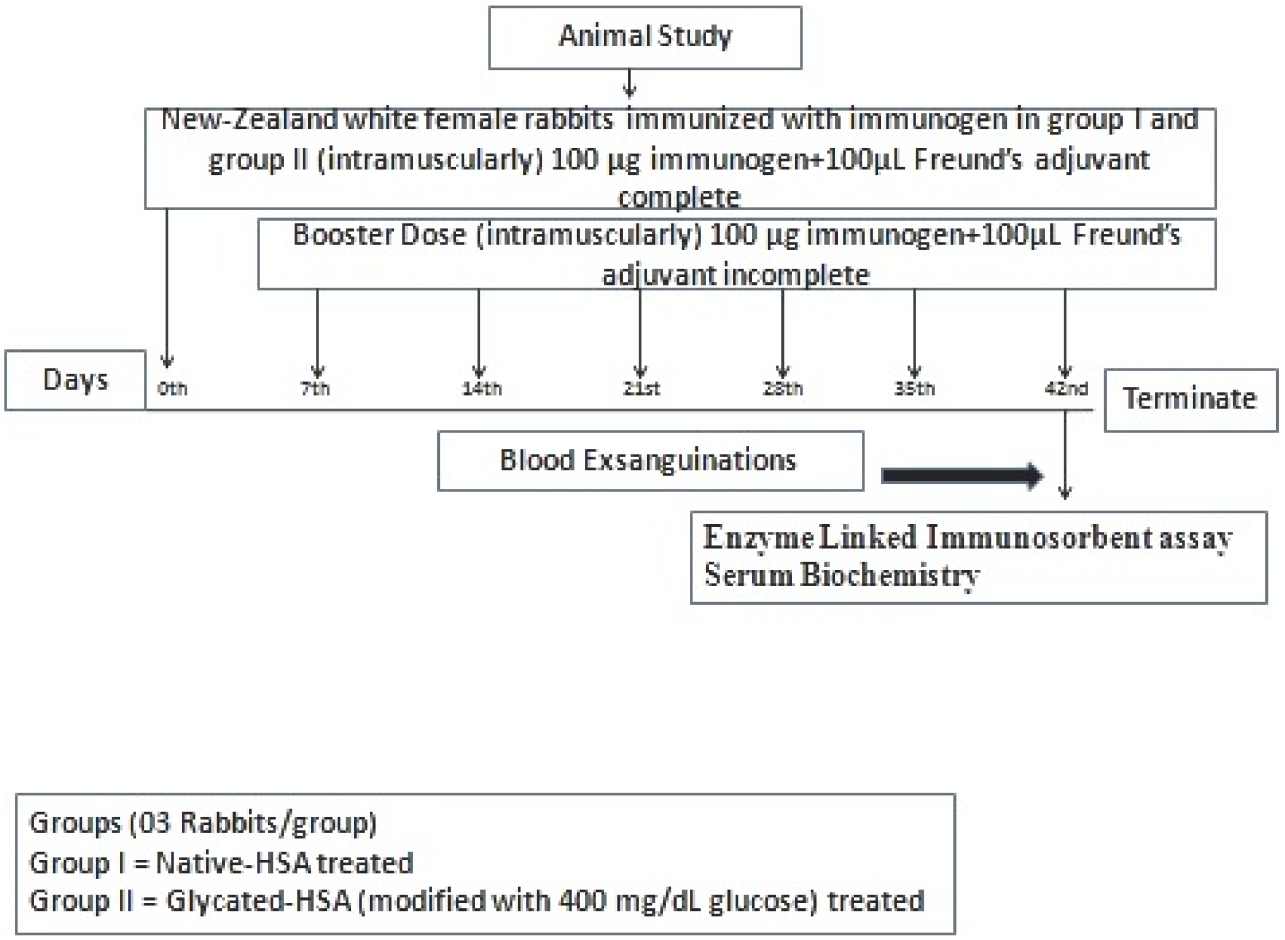
Diagrammatic representation of animal immunization protocol.

The immunization of random, female New Zealand white rabbits (1.5±5 Kgs n = 03/each group) randomly selected and acclimatizedfor one week, performed as in previously described protocol [28]. Immunization was performed intramuscularly at multiple sites with 100 μg each of native and modified protein with 400 mg/dL glucose emulsified with Freund’s adjuvant complete. The rabbits were boosted weekly for 6 weeks with Freund’s incomplete adjuvant with the same amount of protein (immunogen) (Fig 1). Blood was collected from a marginal ear vein, and serum was separated. To inactivate the complement protein serum was given heat treatment at 56°C for 30 min, followed by storage at -20°C in the presence of 0.1% sodium azide as a preservative.

### Haematological and biochemical analysis

Blood sample were collected in K3-EDTA anticoagulant vials for determination of haematological parameters, i.e. white blood cells (WBCs), neutrophils, lymphocytes, monocytes, eosinophils, basophils, red blood cells (RBCs), blood haemoglobin (Hg), hematocrit (HCT), platelets (PLT), mean corpuscular volume (MCV), mean corpuscular haemoglobin (MCH) and mean corpuscular haemoglobin concentration (MCHC) with Sysmex haematological automated analyzer (Sysmex Corporation Lincolnshire, IL 60069).

Sera of rabbits (pre-immune and immune) were used for determination of serum biochemical parameters with Randox biochemical automated analyzer (Randox Laboratories, BT29 4QY, United Kingdom).

### Rectal temperature measurement

The rectal temperature of animals of both groups was recorded with high capacity sensitive digital thermometer at the termination (6^th^ week after immunization) of the experiment.

### Direct binding enzyme-linked immunosorbent assay

ELISA was performed on polystyrene plates with slight modifications [29]. Briefly, 96 wells microtitre plates were coated with 100 μl of native and glucose-modified HSA (10 μg/mL in protein coating buffer) and incubated for 2 hrs at 37°C followed by overnight incubation at 4°C. Each sample was coated in duplicates, leaving half plate devoid of antigen coating that would serve as a control. Unbound antigen was washed thrice with TBS-T followed by blocking with 2.5% fat-free milk in TBS for 4–6 hrs incubation at 37°C. Subsequentlyafter washing thrice with TBS-T, test serum diluted in TBS (100μl/each well) were added. Plates were incubated for 2 hrs at 37°C followed by washing thrice with TBS-T. The bound antibodies were assayed with anti-rabbit/anti-human alkaline phosphatase conjugate followed by incubation at 37°C for 2 hrs. The plates were read at 410 nm after addition of p-nitrophenyl phosphate as substrate.

Results were expressed as themean of A_test_-C_ontrol_. Similarly, ELISA of T2DM, T1DM, GDM and T2DM+CKD patients were performed according to previously describe protocol [29].

### Inhibition ELISA

The immunogenic specificity of the antibody was measured by inhibition ELISA as described previously [30–31]. Briefly, varying amount of inhibitors (native and glucose modified HSA with concentration ranging from 0–20 μg/mL) were incubated with antiserum at 37°C for 2 hrs followed by overnight incubation at 4°C. The immune complex formed thus coated in the wells of polystyrene plate. The steps after the coating were performed same as in direct binding ELISA. The results were expressed using the following equation.

$$\text{Percent Inhibition }=\;1-{\text{ A}}_{\text{inhibited}}/{\text{A}}_{\text{uninhibited}}\times 100$$

Where A _inhibited_ is the absorbance of inhibited wells and A _uninhibited_ is the absorbance of uninhibited wells.

### Isolation of IgG

Serum immunoglobulin G (IgG) from the sera of animals (pre-immune and post-immune) was isolated by affinity chromatography Protein A Agarose column as described by the previous protocol [32]. Briefly, 500μl of serum diluted with an equal volume of PBS (pH = 7. 4, 20 mm) was loaded into the column. The washing cycle of the column was performed 2–3 times to remove unbound IgG. The bound IgG was eluted with previously described protocol [30] followed by neutralization with 1 mL of 1 M Tris-HCl (pH = 8.5). The fractions eluted and read at 251nm and 278 nm. IgG concentration was determined (1.40 OD280 = 1.0 mg/mL) and dialyzed against PBS (pH = 7.4) and stored -20°C with 0.1% sodium azide.

### Direct binding and inhibition ELISA of purified IgG from an animal model

The direct binding and inhibition ELISA of isolated IgG were performed in a microtitre plate as described previously [30–31].

### Spectroscopy of antigen-antibody complexes

The ultraviolet absorption spectra of serum-antigen (HSA native and glucose modified) and isolated rabbit IgG-antigen (HSA native and glucose modified) in the form of the immune complex were recorded in the range of 233–450 nm. Briefly, the immune complex was formed upon incubation of antigen (native and glucose modified HSA with concentration ranging from 0–20 μg/mL) with isolated IgG for 2 hrs at 37°C followed by overnight incubation at 4°C. Fluorescence profile of immune complexes formed was scanned with an excitation wavelength of 278 nm and emission range of 450–550 nm.

### X-ray diffraction of immune complex to determine epitopes

To determine the immune complex formation, i.e. presence of epitopes to bind to the antigen (both native and glucose modified HSA), the glycated albumin (antigen) with a concentration of 10μg incubated with isolated IgG (20μM) from animal sera (immunized with native HSA and glucose modified HSA) for 2 hrs at 37°C. An x-ray diffraction pattern of the film was recorded with Siemens D 5000 diffractometer installed with a flat monochromator. The divergence of the primary beam was enough to permit the low, glancing angles of incidence. Step-scan mode with increments of 0.020 2θ with the count for 1s at each step was selected for recording the data. The angle of incidence α was set at a range of 0.20 to 100 to vary the interaction diameter and length in each depth area/region with the final scanning of the exit angle.

### Statistical analysis

Data are given as mean ± SD. The statistical significance of the data was determined by student’s t-test (stat graphics, origin 6.1). A p value of <0.05 was considered statistically significant.

## Results and discussion

### Spectroscopic analysis

HSA (20μM) was incubated with varying concentrations (100, 200, 300 and 400 mg/dL) of glucose under sterile conditions for 40 days at 37°C and UV absorption spectra were recorded. Native HSA gave a characteristic peak at 280 nm, whereas glucose modified samples of HSA showed an increasing % hyperchromicity. Marked increase in % hyperchromicity at λ_280 nm_was recorded with highest modified HSA sample as compared to native HSA. Glucose modified HSA showed 65.82%, 71.98%, 73.62% and 76.63% hyperchromicity with increasing glucose concentration at λ_280 nm._ The change in absorbance may be attributed to the formation of AGEs and their aggregates resulting from cross-linking[32–33]. Glycation of HSA protein enables the aromatic amino acids to expose their cyclic ring structure electrons that contribute to hyperchromicity phenomenon. Apart from aromatic amino acids, the unfolding of globular structure and reshuffling of bonds and their electrons also contribute to this effect [34–35].

Possible formation of fluorogenic AGEs was measured from the specific emission fluorescence intensity observed at 370 nm excitation wavelength. Native HSA at this excitation wavelength showed negligible changes fluorescence. Under identical and controlled experimental conditions, the gradual increase in fluorescence intensity of glycated-HSA samples indicates the formation of pentosidine like fluorogenic AGEs [36]. The marked increase in fluorescence intensities observed in glycated samples were 23.82%, 35.69%, 42.87%, and 68.22% compared to native HSA.

**Fig 2:**
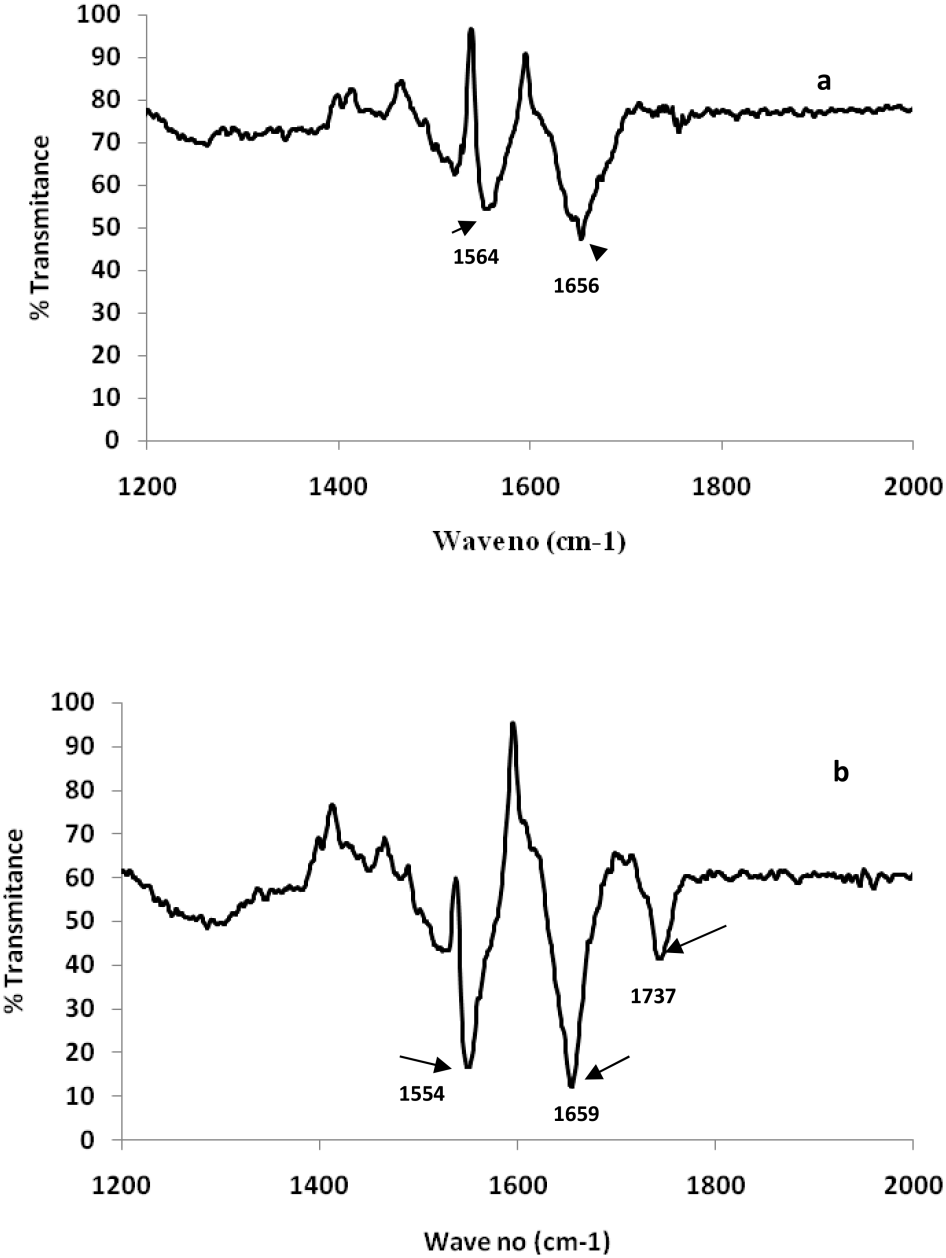
FTIR spectra of (a) native HSA (b) HSA modified with 400 mg/dL of glucose.

FTIR spectra of both native and glycated-HSA with 400mg/dLwere recorded as shown in Fig 2a and 2b respectively to demonstrate the alterations in thesecondary structure on the basis of frequency and shapes of amide I and amide II bands.Amide I showed a characteristic peak at 1656 cm^-1^ in native HSA that showed shifting to 1659 cm^-1^ in glycated-HSA that contributed in theabsorption peak of α-helix. Similarly, amide II band (N-H bend vibrations of peptide bonds) showed shifting at 1554 cm^-1^ in glycated-HSAcompared to 1564 cm^-1^ of native. A new peak at 1737 cm^-1^ corresponding to thealdehyde group of carbonyl compound was observed inglycated–HSA. Moreover, in native HSA this peak was absent.

FT-IR spectral results clearly demonstrated the changes in the secondary structure of HSA on post glucose modifications that contributed to band intensity and position displacement. Previous literature showed the presence of variable vibrational peptide moieties in amide bands of proteins [36]. Amide I and II of protein primarily contribute as two major signature bonds in theinfra-red region. Amide I band intensity corresponds to α-helix associated C = O vibrational stretching, while amide II relates with N-H and C-N [29]. The results of the present study also favoured the findings of previous literature on glycation induced alterations in HSA secondary structures [36].

### Liquid chromatography mass spectroscopic analysis

**Fig 3:**
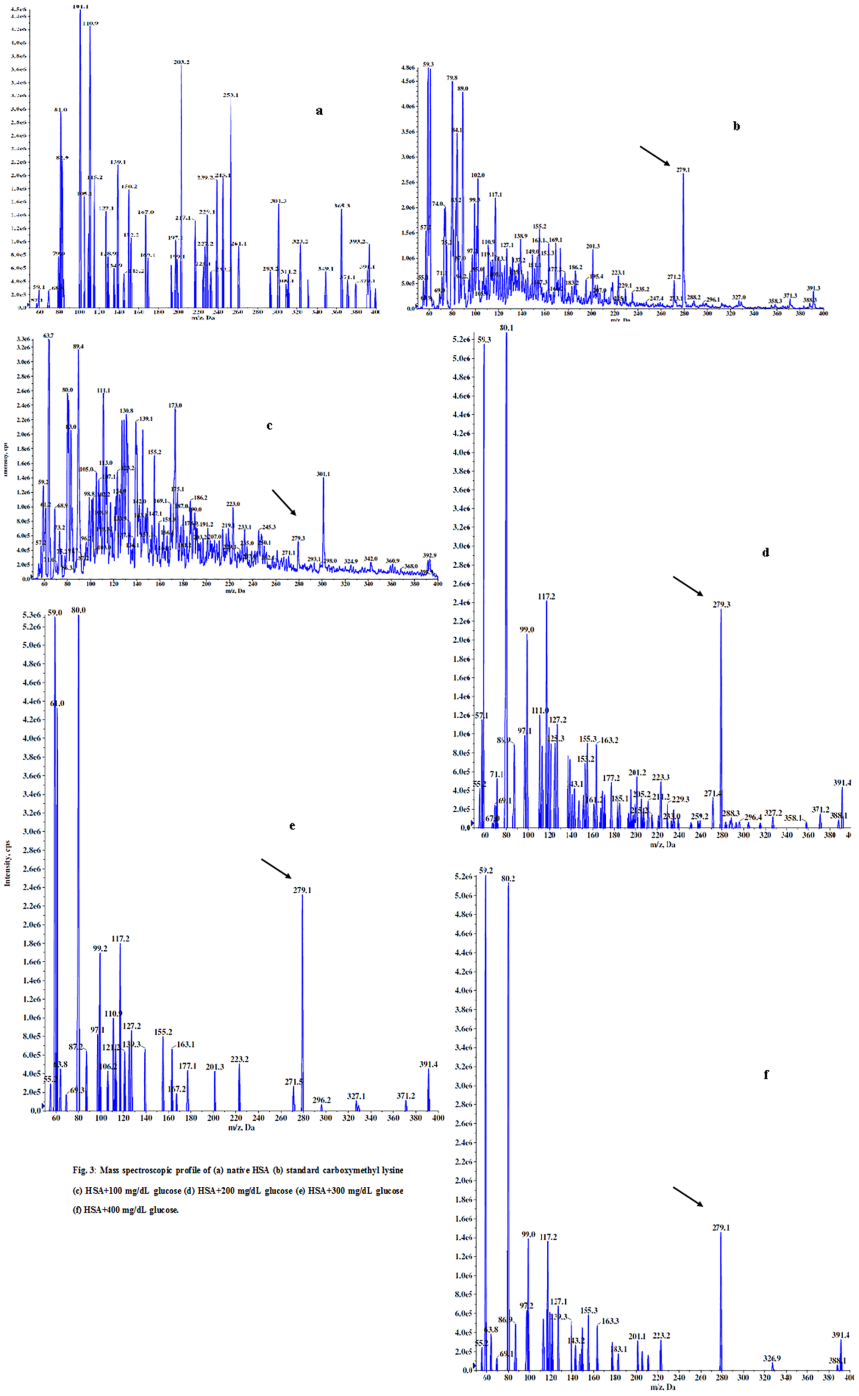
Mass spectroscopic profile of (a) native HSA (b) standard carboxymethyl lysine (c) HSA+100 mg/dL glucose (d) HSA+200 mg/dL glucose (e) HSA+300 mg/dL glucose (f) HSA+400 mg/dL glucose.

Carboxymethyl-lysine (CML) a, known gold standard of advanced glycation end product (AGEs) was used to confer the formation of AGEs in glycated-HSA samples with the LCMS coupled with reverse phase UPLC system. Fig 3a–3f shows themass spectroscopic profile of standard CML, acid hydrolyzed native HSA (unmodified) along with acid hydrolyzed glycated-HSA (modified with D-glucose). As shown in Fig 3a–3f showed an m/z value of 279.1 in all glycated samples matching with the standard CML. No such species were observed in hydrolyzed native HSA mass spectra.

### Haematological and biochemical analysis

Hematologic values along with serum biochemical parameters were shown in Tables 1 and 2 respectively.

**Table 1:** Heamtological and rectal temperature analysis of preimmune and immune rabbits demonstrating the inflammatory response after immunization with glycated HSA (antigen).

Groups/Parameters	WBCS	RBCS	NEUT	LYMPH	MONO	EO	BASO	PLATE	Rect. Temp.(F)
**Pre Immune (n = 3)**	7.85±0.71	5.57±1.03	4.95±0.54	1.93±0.17	0.07±0.02	0.04±0.02	0.03±0.01	240±50.23	100.07±0.75
**Post Immune (n = 3)**	9.31±0.84*	5.08±0.29	6.16±0.24	2.72±0.32	0.29±0.00	0.09±0.01	0.11±0.03	470±45.21*	104.33±0.87

The data are represented as mean±S.D and p value <0.05 considered to be significant shown by *. Abbreviation used: WBCs = White blood cells, RBCs = Red blood cells, NEUT = Neutrophils, LYMPH = Lymphocytes, EO = Eosinophils, BASO = Basophils, PLATE = Platellets,Rect. Temp. (F) = Rectal temperature in Fahrenheit.

**Table 2:** Biochemical analysis of preimmune and immune rabbits after immunization with glycated HSA (antigen).

Groups/Parameters	CRT	ALB	ALT	AST	ALKP	TBIL	UREA	URIC ACID	TPROT
**Pre Immune (n = 3)**	0.56±0.05	2.20±0.16	21.00±2.00	19.33±1.52	25.33±2.51	0.06±0.05	24.33±3.05	0.10±0.00	3.06±0.15
**Post Immune (n = 3)**	1.10±0.18	3.58±0.10	37.33±6.42	25.33±2.51*	74.66±20.81*	0.37±0.06	44.00±4.35	0.13±0.05	5.70±0.36

The data are represented as mean±S.D and p value <0.05 considered to be significant shown by *. Abbreviation used: CRT = Creatinine (mg/dL), ALB = Albumin (g/dL), ALT = Alanine Transferase (U/L), AST = Aspartate trans cabamylase (U/L), ALKP = Alkaline Phosphatase (U/L), TBIL = Total Bilurubin (mg/dL), TPROT = Total Protein (g/dL), UREA = Urea (mg/dL), URIC ACID = Uric Acid (mg/dL).

### Rectal temperature measurement

Rectal temperature of rabbits immunized with native HSA (unmodified) and glycated-HSA (modified with D-glucose 400 mg/dL) were recorded (Table 1) and showed arise in temperature in rabbits immunized with glycated-HSA compared to native. The immunogenicity and inflammatory response of AGEs have been studied extensively in the present study. The inflammatory response is mediated by receptor for AGE (RAGE), a prime molecule in the activation and initiation of differentiation of monocytes on the cell surface upon binding with AGEs that mediate the release of pro-inflammatory cytokines along with chemokines. Macrophages are present in varied tissues that upon activation with pathogen recognition or pro-inflammatory molecules represent with extreme phagocytic mechanisms. Literature has supported that macrophage expresses RAGE receptors similar to circulating monocytes [37]. The immunization of rabbits with *in-vitro* formed AGEs triggers the pro-inflammatory immune responses mediated by expression of immune cells (WBCs, Eosinophils, Basophils, and Neutrophils) as shown clearly from the results of the present study (Table 1). A recent literature favors the results of this study by demonstrating the role of RAGE in diabetes mellitus and the immune response [38]. Ann Marie Schmidtproposed “two-hit” model for vascular perturbation for RAGE upon binding with its ligands [39]. The “first hit” devotes to enhanced RAGE expression in the presence of its associated ligands,whilst “the second hit” occurs in the presence of diverse stress actions along with external factors that exacerbatepro-inflammatory responses. The Inflammatory potential of injected immunogen is not perturbing the serum biochemistry of animals, thereby showing no significant changes (Table 2).

### Serum direct binding and inhibition ELISA

**Fig 4:**
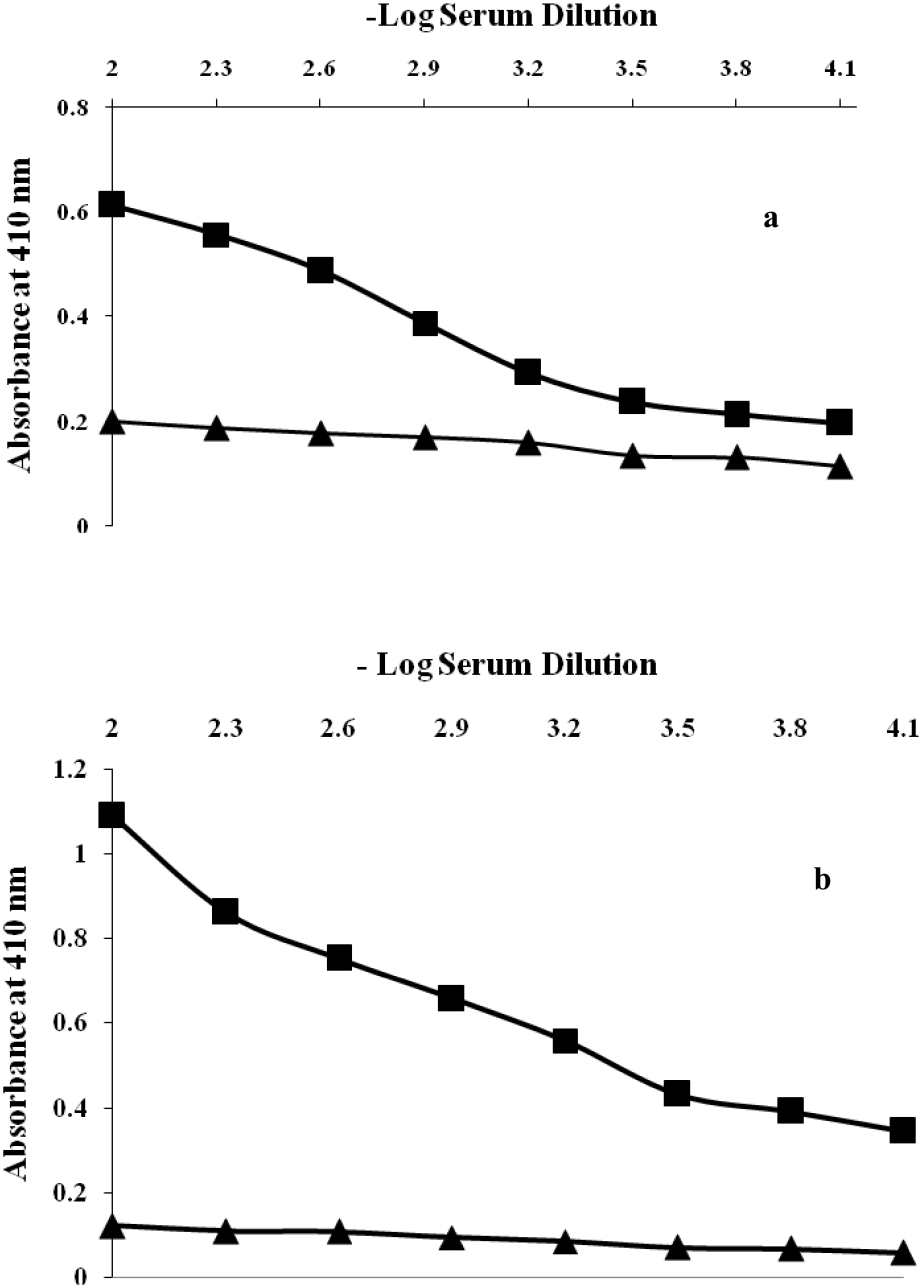
(a) Level of induced antibodies against native HSA. Direct binding ELISA of native HSA with pre-immune and immune sera. The microtitre plates were coated with native HSA (10 μg/mL). (b) Level of induced antibodies against glucose modified HSA. Direct binding ELISA of glucose modified HSA with pre-immune and immune sera. The microtitre plates were coated with native HSA (10 μg/mL).

Native HSA (antigen) immunized rabbits showed moderately antibody immune response with a classical antibody titre of <1:6400 as demonstrated by serum direct binding ELISA (Fig 4a). On the other hand,glycated-HSA was proved to be a potent immunogen with potential to provoke antibodies generation with ahightitre of >1:12,800 (Fig 4b). Under identical conditions pre-immune serum of animals immunized with both native and glucose modified antigen showed negligible binding titre.

**Fig 5:**
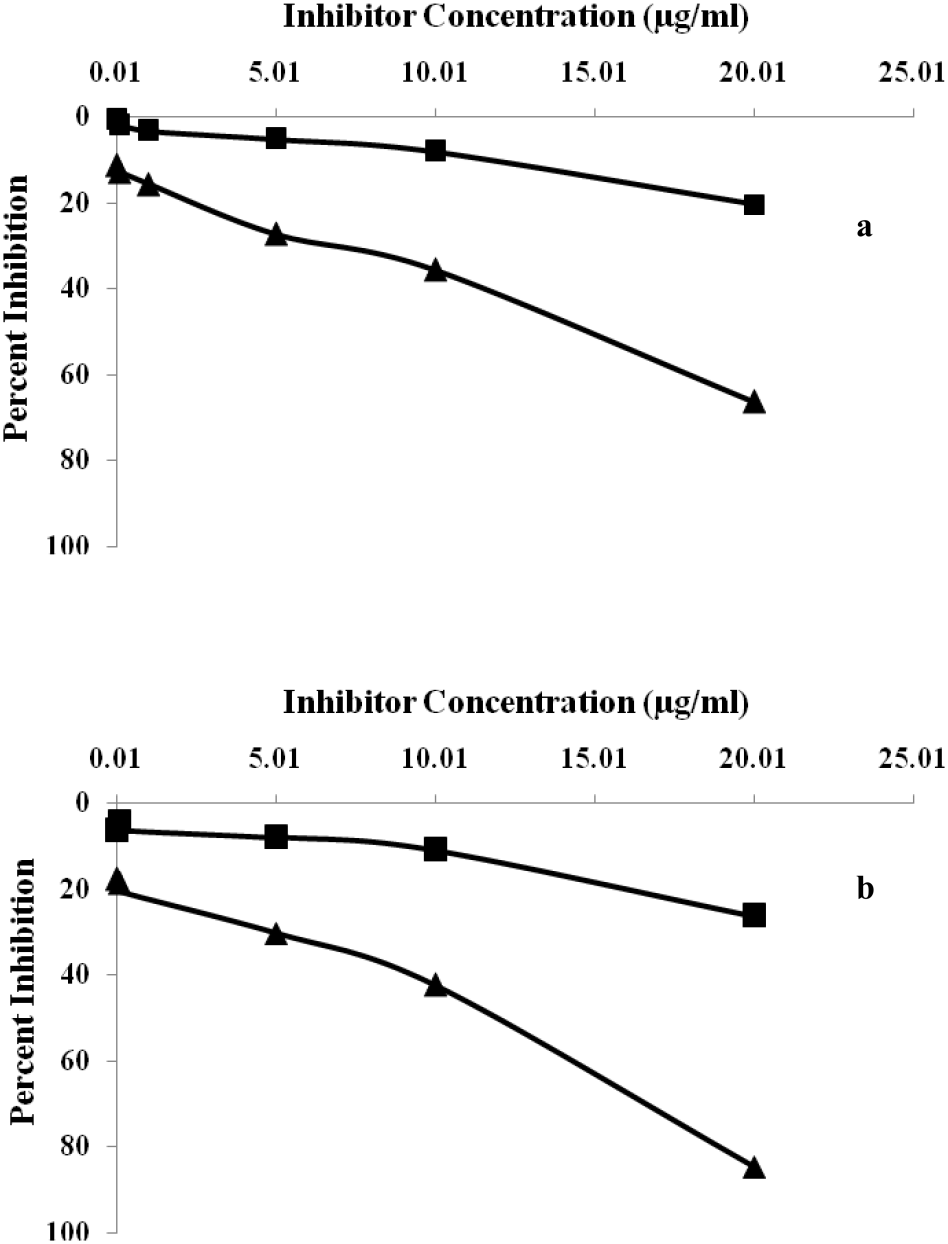
(a) Inhibition ELISA of anti-HSA immune and pre-immune sera with native HSA. Microtitre plates were coated with native HSA (10 μg/mL). (b) Inhibition ELISA of anti-glucose-HSA immune and pre-immune sera with glucose modified HSA. Microtitre plates were coated with native HSA (10 μg/mL).

The specificity of induced antibody titre in the serum of immunized rabbits was evaluated by competition inhibition ELISA. Immunized rabbits with glycated-HSA (modified with 400 mg/dL D-glucose) showeda classical inhibition of 84.65% with an antigen concentration of 20μg/mL (Fig 5a). Induced antibodies raised against native-HSA (immunogen) showed a maximum inhibition of 66.6% at same immunogen concentration (Fig 5b). Under identical conditions pre-immune serum of animals immunized with both native and glucose modified antigen showed negligible binding titre.

In the present study, immunogenicity of native and glycated—HSA was examined by inducing antibodies in female rabbits. A rabbit immunized with glucose modified—HSA possesses high serum antibody titer in direct binding ELISA compared to native. The results of thepresent study demonstrate that high antibodies titer in rabbits immunized with glycated-HSA represents structural impairments on HSA structure, leading to the generation of neo-epitopes, rendering them with more immunogenic potential. Similarly, high percent inhibition was observed in sera of rabbits immunized with glucose modified antigen. Prolonged incubation of HSA with D-glucose exhibits more gluco-oxidation and formed neo-epitopes due to its folding and unfolding of theglobular structure. Antigenicity of affinity purified anti-glycated-HSA IgG repeatedly states that purified IgG preferentially recognizes the neo-epitopes formed due to glucose-induced modifications thereby showing maximum percent inhibition. Native HSA upon recognition with affinity purified IgG shows less percentinhibition. A previous interpretation was publishedthat demonstrated the formation of auto-antibodies against gluco-oxidatively modified HSA [39]. The results of the present study are in full agreement with earlier reported results of ageneration of auto-antibodies against glycated-HSA at higher concentration of glucose [40].

### Direct binding and inhibition ELISA of rabbit isolated IgG

**Fig 6:**
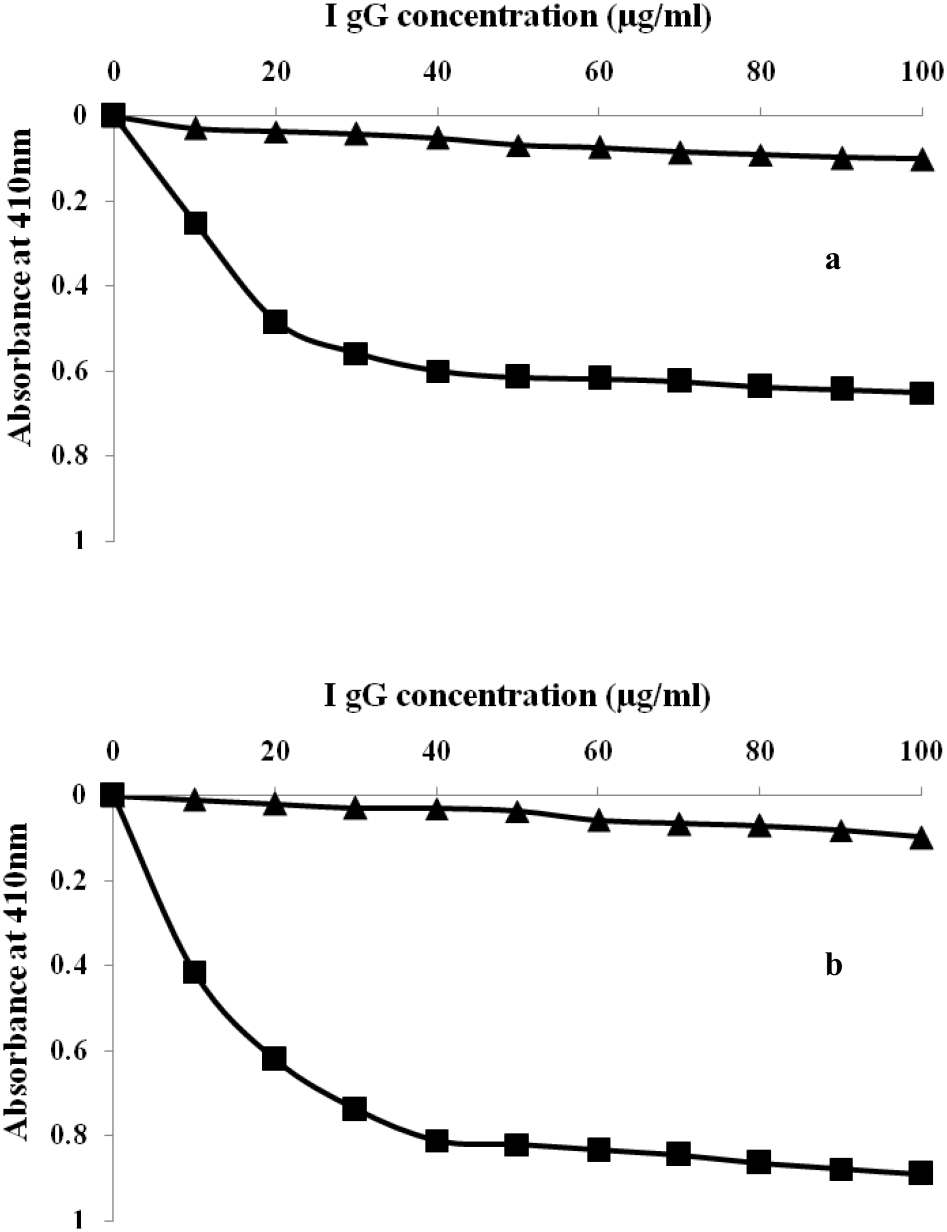
(a) Binding of affinity purified anti-HSA IgG and pre-immune IgG to native HSA. Microtitre plates were coated with native HSA (10 μg/mL). (b) Binding of affinity purified anti-glucose modified-HSA IgG and pre-immune IgGto glucose modified- HSA. Microtitre plates were coated with glucose modified- HSA(10 μg/mL).

**Fig 7:**
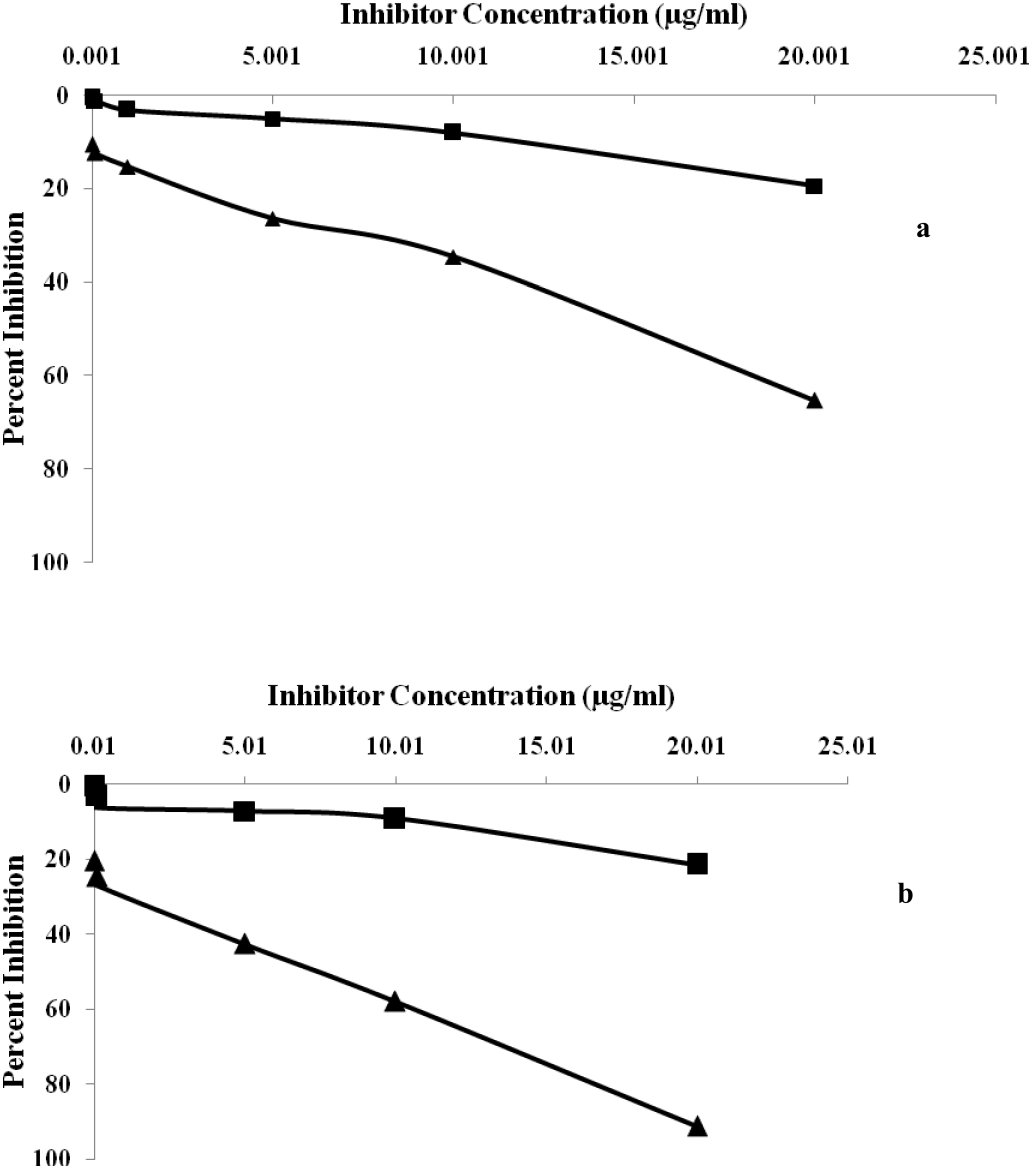
(a) Inhibition of binding of affinity purified anti-HSA IgG and pre-imune IgG to native HSA. Microtitre plates were coated with native HSA (10 μg/mL). (b) Inhibition of binding of affinity purified anti-glucose-HSA IgG and pre-imune IgG to glucose modified HSA. Microtitre plates were coated with native HSA (10 μg/mL).

IgG purified from the serum of pre-immune and immune rabbits were subjected to direct binding and competitive inhibition ELISA on microtitre plates coated with native and glycated-HSA. Direct binding ELISA of purified IgG showed strong binding with their respective immunogens (Fig 6a and 6b). The saturation of native and glycated-HSA was obtained at 40 and 60 μg/mL of IgG concentration respectively. However, pre-immune IgG showed negligible binding under identical conditions. The specificity of epitopes present at native and glycated-HSA towards their respective antibody was characterized by inhibition immunoassay. A value of 91.23% inhibition was achieved upon binding with glucose modified albumin (Fig 7a). Inhibition of 65.23% in antibody binding at 20μg/mL was achieved with native HSA (Fig 7b).

### Serum direct binding ELISA in patients with diabetes mellitus

**Fig 8:**
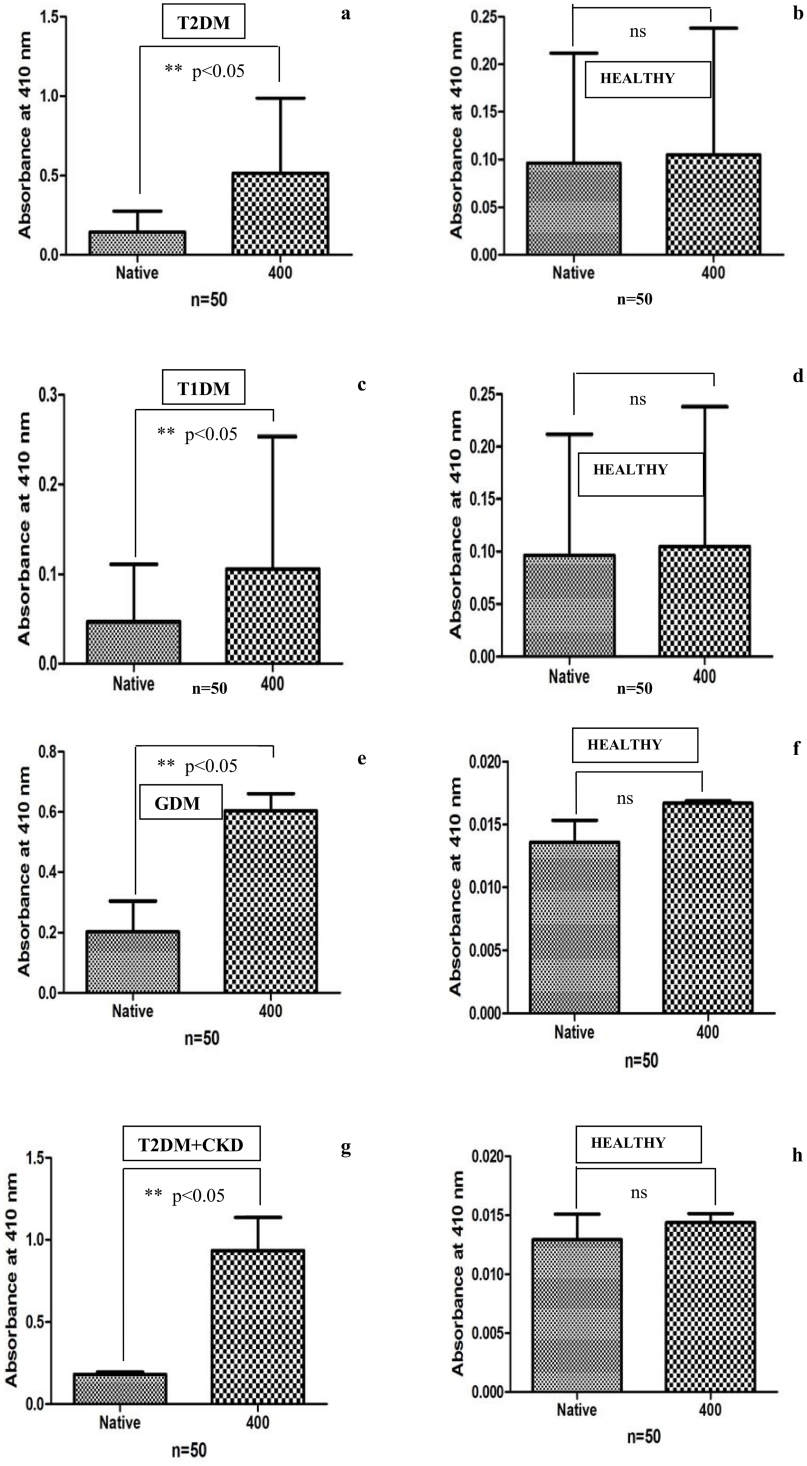
Direct binding ELISA of serum auto-antibodies in (a) T2DM (n = 50) (c) T1DM (n = 50) (e) GDM (n = 50) (g) T2DM+CKD (n = 50) with native and 400 mg/dL glucose modified HSA and normal human sera (NHS) served as control in (b) (n = 50), (d) (n = 50), (f) (n = 50) and (h) (n = 50). The plate was coated with the respective antigens (10μg/mL). A p value <0.05 considered to be significant. ns = non-significant

Auto-antibodies against glycated-HSA in diabetic sera were detected by ELISA on microtitre plate coated with native and glycated- HSA (modified with 400 mg/dL glucose). The bindingtitre of T2DM, T1DM, GDM and T2DM+CKD diabetic sera with native and glycated-HSA havebeen shown in Fig 8. Auto-antibodies in diabetic subjects showed more binding affinity with glycated-HSA (p<0.05) compared to native HSA. Sera of healthy subjects included as acontrol did not show significant binding with the coated antigens (10μg/mL).In thecontext of theimmunogenicbehaviour of AGEs, the present study was aimed to suggest the antibodies generation against AGEs formed at even low glucose concentration (400 mg/dL) that may be reported in diabetic subjects with complications. The *in-vivo* generation of auto-antibodies against glycated-HSA can be explainedregardingprolonged poor glycemic control that may induce the structural modifications in HSA generating neo-epitopes against which antibodies are raised in serum. In previous studies association between glycated-HSA and immunoglobulin heavy chain, constant regions havebeen described several times [41–43]. An elevated level of auto-antibodies in T1DM subjects due to increased content of fructosamine was found compared to T2DM patients [44]. A recent study focused on the role of AGEs in diabetes-associated complications [45]. Sakai et al. studied the production of auto-antibodies in subjects with nephropathy against glycated-HSA [46]. The present work showed the generation of auto-antibodies in diabetic subjects with type 2, type 1, gestational diabetes and type diabetes subjects with chronic kidney disease and found increased binding of antibodies against glycated-HSA coated wells compared to native HSA due to neo-epitopes formed as a resultant of structural modifications upon glycation. Similar results havebeen shown with ananimal model in thepresent study. Normal human sera showed negligible binding with anantigen.

### Spectroscopy of antigen-antibody complexes

**Fig 9:**
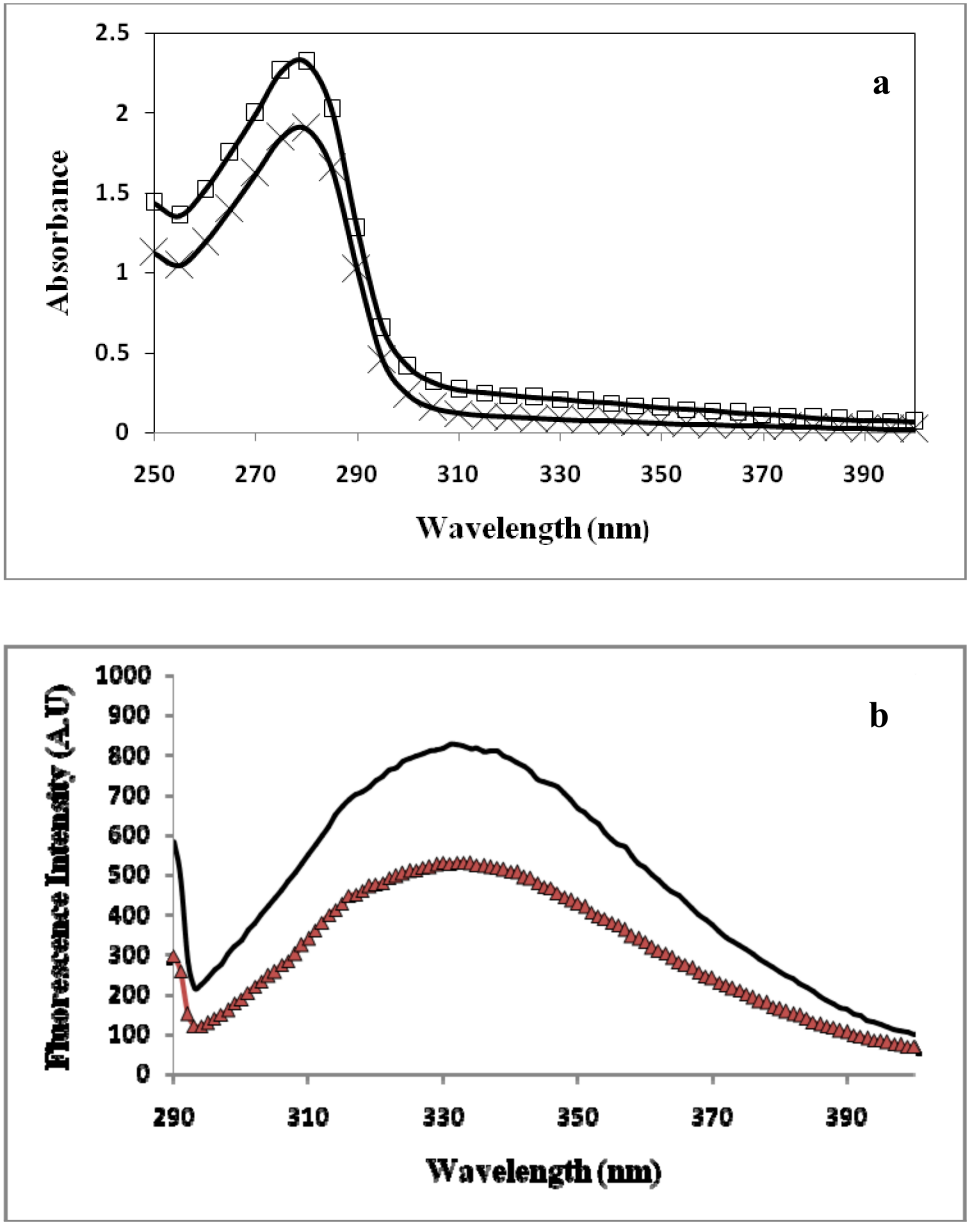
(a) Ultraviolet absorption spectra of native HSA+IgG immune complex and AGEs HSA+IgG immune complex with 400 mg/dL D-glucose. Fluorescence emission profile of immune complex (native HSA+IgG) shown by black colour and immune complex (AGE-HSA+IgG) shown by red colour.

The UV-absorption profile of isolated IgG and immune complexes (i.e., native HSA-IgG and glycated-HSA-IgG) were recorded in a wavelength range of 200–400 nm as shown in Fig 9a. The Decrease in absorption was reported in immune complexes as compared to UV spectra of IgG alone. Intrinsic fluorescence of immune complexes along with isolated IgG alone was recorded as shown in Fig 9b, demonstrated that immune complexes show quenching in fluorescence intensity compared to thefluorescence intensity of isolated IgG alone. The reason behind the maximum absorption of IgG alone is the availability of all bonding and aromatic amino acids constituting epitopes showing absorbance in UV region. Immune complex with native HSA-IgG showed less absorbance compared to IgG alone due to structural changes and engagement of epitopic residues and bonding electrons in binding. Immune complex formed between glycated-HSA-IgG showed maximum hypochromicity due to extensive structural modifications that lead to thegeneration of neo-epitopes, which upon bindingwith antigen decreases UV absorption mediated by stearic hindrance.

Fluorescence of immune complex also clearly demonstrates the formation of neo-epitopes in an immune complex that exhibits intrinsic quenching of tyrosine and tryptophan compared to IgG alone and native immune complex. Neo-epitopes in glycated-HSA- immune complex showed stearic hindrance and change in microenvironments of these aromatic amino acids present in the vicinity of epitopes, contribute in this quenching and demonstrate the pattern as IgG alone>HSA-IgG>HSA glucose modified-IgG (Fig 9b). The glucose concentration used in this experiment is low and therefore, no correction of inner filter effect inner filter effect in fluorescence spectra measurement. A work performed on molten globule states of cytochrome C showed that low concentration of N-alkyl sulphates alters its electrochemical behaviour [47]. The same property has also been studies in HSA upon non enzymatic glycation [23]. Another study done by Ahmadi et al. In 2014, uses fluorescence, CD and UV spectroscopic techniques to demonstrates the complex formation between β-lactoglobulin and retinol at interplay of hydrophobic interactions [48]. Previous study uses spectroscopic approach to demonstrate the interaction between human serum albumin and carbonyl cyanide p- (trifluoromethoxy) phenylhydrazone (FCCP) [49]. In another study done on HSA interaction, fluorescence spectroscopy and zeta potential was used to decipher complex formation [50].

### X-ray diffraction of immune complex to determine epitopes

**Fig 10:**
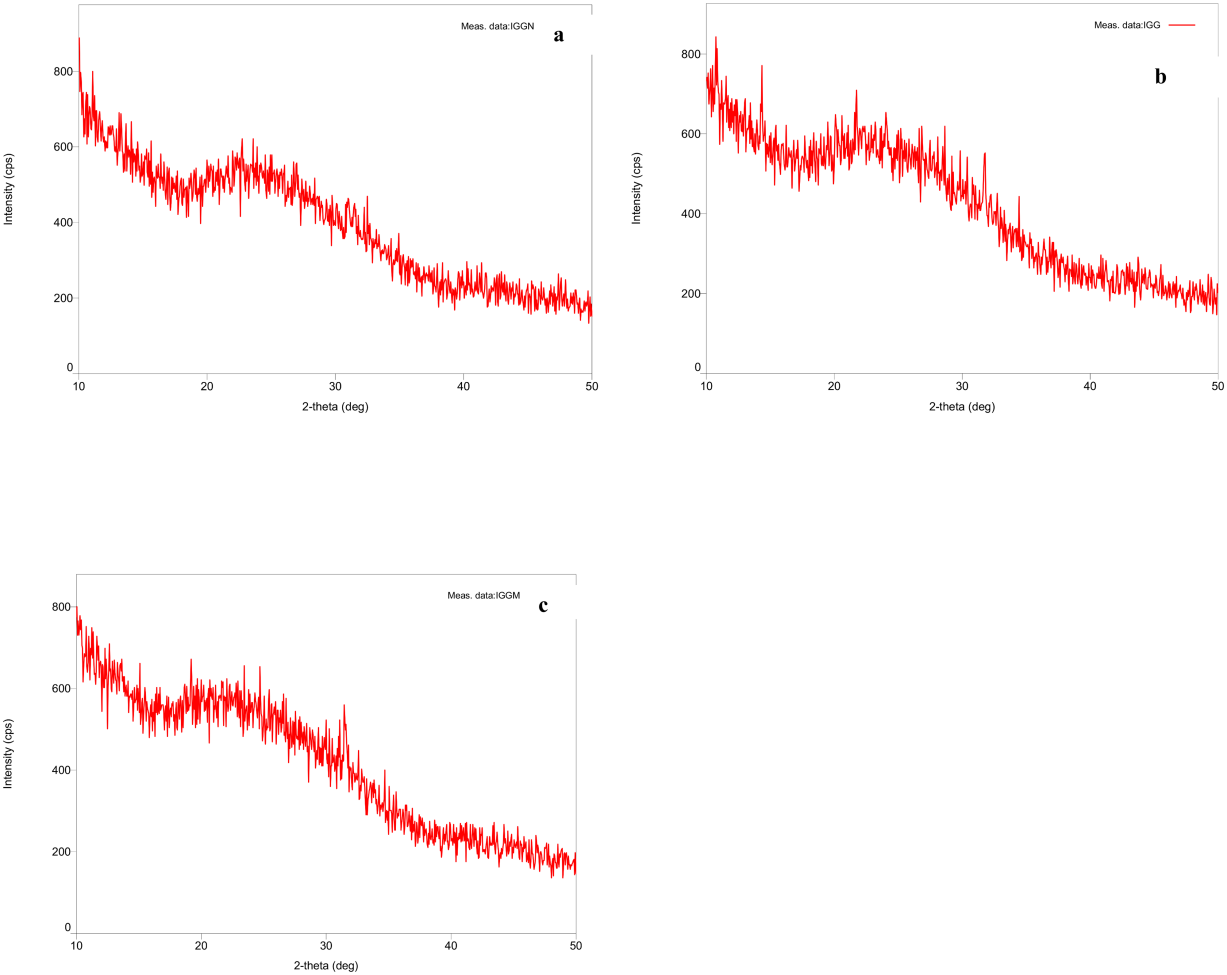
X-ray diffraction pattern under scan range of 10–500 (a) affinity purified IgG alone (b) immune complex of native HSA with IgG (c) immune complex of AGE-HSA (modified with 400 mg/dL D-glucose) with IgG.

X-ray angular diffraction pattern of affinity purified IgG along with immune complexes (i.e. native HSA-IgG and glycated-HSA-IgG) were recorded with flat monochromator on a slide containing afilm of the same. Fig 10a–10c clearly demonstrates the alteration patterns in the immune complexes upon compared to isolated IgG alone. Fig 10a shows the Braggs peaks of affinity purified IgG film with an angular value of 9.97E+00. Fig 10b and 10c show the distortion in the structure of the isolated IgG due to binding of epitopes with the native and glycated-HSA antigen thereby showing Braggs peak shifting in the diffractogram with a value of 1.93E+00 and 1.35E+01 respectively.Several published literature revealed the structure of HSA by small-angle X—ray scattering [51], quasi—elastic light scattering [52], hydrodynamic technique [53], neutron scattering [54] and 1-H NMR [55] but surprisingly X-ray diffraction pattern of the immune complex formed between affinity purified IgG with their respective antigens has not yet solved. Here, thefirst time we describe the x-ray diffraction pattern of affinity purified IgG along with immune complex to investigate the formation of the new epitopes generated as a resultant ofglucose modifications on HSA. The thin film diffractogram of IgG alone showed the Braggs angular diffraction of 9.97E+00 while theimmune complex with native and glucose modified antigen showed angular diffraction of 1.93E+00 and 1.35E+01 respectively, therebyconferring the structural alterations of these bio-molecules rendering generation of neo-epitopes due to glycation (Fig 9a–9c). The attemptto identify and characterize the immune complex with these novel techniques to prove the formation of glycation induced neo-epitopeshas been implicated well and furthermore extensive studies are required in this approach.

## Conclusion

Our study revealed extensive binding of circulating auto-antibodies with D-glycated-HSA in comparison with its native form indicating the fact that glucose-induced structural modifications in HSA molecule represent the structural compactness and generation of neo-epitopes on the protein. The neo-epitopes render the HSA antigenic potential, due to the distortion of HSA structure upon glycation that formed auto-antibodies against self-protein. The direct binding ELISA and inhibition ELISA also validate the formation of neo-epitopes. Furthermore, the immune complex formed between induced antibodies and protein complex thereby represents the formation of neo-epitopes formed that havebeen further revealed with spectroscopic and X-ray diffraction pattern.

## Limitation of the study

The obvious limitation of the present study is the range of glucose selected for the modifications. Due to near similar range ofD-glucose concentrations used in this study (i.e 100 mg/dL to 400 mg/dL), the experiments were performed with only native (unmodified) and glycated-HSA (modified with 400 mg/dL glucose).
